# Lysophosphatidic Acid Induces Podocyte Pyroptosis in Diabetic Nephropathy by an Increase of Egr1 Expression via Downregulation of EzH2

**DOI:** 10.3390/ijms24129968

**Published:** 2023-06-09

**Authors:** Donghee Kim, Ka-Yun Ban, Geon-Ho Lee, Hee-Sook Jun

**Affiliations:** 1 Lee Gil Ya Cancer and Diabetes Institute, Gachon University, Incheon 21999, Republic of Korea; 2 College of Pharmacy and Gachon Institute of Pharmaceutical Sciences, Gachon University, Incheon 21936, Republic of Korea; qksrkdbs@naver.com (K.-Y.B.); univer3768@naver.com (G.-H.L.); 3 Gachon Medical Research Institute, Gil Hospital, Incheon 21565, Republic of Korea

**Keywords:** lysophosphatidic acid (LPA), podocyte, pyroptosis, Egr1, EzH2

## Abstract

Podocyte damage and renal inflammation are the main features and pathogenesis of diabetic nephropathy (DN). Inhibition of lysophosphatidic acid (LPA) receptor 1 (LPAR1) suppresses glomerular inflammation and improves DN. Herein, we investigated LPA-induced podocyte damage and its underlying mechanisms in DN. We investigated the effects of AM095, a specific LPAR1 inhibitor, on podocytes from streptozotocin (STZ)-induced diabetic mice. E11 cells were treated with LPA in the presence or absence of AM095, and the expression of NLRP3 inflammasome factors and pyroptosis were measured. A chromatin immunoprecipitation assay and Western blotting were performed to elucidate underlying molecular mechanisms. Gene knockdown by transfecting small interfering RNA was used to determine the role of the transcription factor Egr1 (early growth response protein 1) and histone methyltransferase EzH2 (Enhancer of Zeste Homolog 2) in LPA-induced podocyte injury. AM095 administration inhibited podocyte loss, NLRP3 inflammasome factor expression, and cell death in STZ-induced diabetic mice. In E11 cells, LPA increased NLRP3 inflammasome activation and pyroptosis via LPAR1. Egr1 mediated NLRP3 inflammasome activation and pyroptosis in LPA-treated E11 cells. LPA decreased H3K27me3 enrichment at the Egr1 promoter in E11 cells by downregulating EzH2 expression. EzH2 knockdown further increased LPA-induced Egr1 expression. In podocytes from STZ-induced diabetic mice, AM095 suppressed Egr1 expression increase and EzH2/H3K27me3 expression reduction. Collectively, these results demonstrate that LPA induces NLRP3 inflammasome activation by downregulating EzH2/H3K27me3 and upregulating Egr1 expression, resulting in podocyte damage and pyroptosis, which may be a potential mechanism of DN progression.

## 1. Introduction

With the growing prevalence of diabetes worldwide, the prevalence of diabetic nephropathy (DN), a major microvascular complication of diabetes, is also increasing [1,2]. Diabetes induces structural changes in the kidneys, including thickening of the glomerular basement membrane, mesangial matrix expansion, the fusion of podocyte foot processes, and loss of podocytes [3]. Podocytes are highly specialized epithelial cells located in the outermost layer of the glomerulus [4], and intracellular oxidative stress and inflammatory responses are pathophysiological mechanisms mediating podocyte damage [5].

Lysophosphatidic acid (LPA) is a small bioactive phospholipid that consists of a glycerol backbone with a hydroxyl group, a phosphate group, and a fatty acid chain [6,7]. It is well-established that LPA is present in all mammalian cells and tissues, including blood, and can function and mediate critical responses during both developmental and pathological conditions by interacting with six specific G-protein-coupled transmembrane receptors (GPCRs; LPA receptors [LPAR] 1–6) [8,9]. Levels of LPA and its precursor, lysophosphatidylcholine (LPC), were elevated in the renal glomeruli of diabetic mice [10]. Moreover, patients with diabetes experiencing DN had significantly enhanced urinary levels of LPA and LPC when compared with patients without DN [11]. Additionally, several previous studies, including by our research group, have shown that inhibition of LPA signaling can attenuate DN-associated fibrosis and renal inflammation in diabetic animal models [12,13,14,15]. Although the antagonism of LPAR1/3 protects against podocyte loss in endothelial nitric oxide synthase (eNOS)^−/−^ db/db mice, a type 2 diabetes model [15], the role of LPA/LPAR1 signaling in podocyte damage during DN remains unexplored.

AM095 is an orally bioavailable LPAR1-specific antagonist [16]. Administration of AM095 at 30 mg/kg in mice suppressed renal fibrosis following unilateral ureteral obstruction without any effects in the sham group and inhibited dermal fibrosis in a scleroderma mouse model [16,17]. Recently, both preventive and therapeutic administration of AM095 improved hypertensive renal damage in Dahl–Iwai salt-sensitive rats [18]. In addition, we previously reported that administration of AM095 improved DN in streptozotocin (STZ)-induced diabetic mice by suppressing Toll-like receptor (TLR) 4/NF-κB signaling-mediated inflammation in mesangial cells [13].

High glucose and advanced glycation end-products (AGEs) can induce renal inflammation, which contributes to the pathogenesis and progression of DN [19,20]. Renal inflammation during DN exacerbates renal damage by inducing sterile inflammation via NLRP3 inflammasome activation in endothelial cells and podocytes [18]. In patients with DN and relevant animal models, the expression of NLRP3 inflammasome activation markers, including cleaved-caspase 1 and interleukin (IL)-1β, was found to be significantly increased in glomeruli [21,22]. In particular, hyperglycemia in streptozotocin (STZ)-induced or db/db diabetic mice can induce NLRP3 inflammasome activation in glomerular podocytes, resulting in podocyte loss and albuminuria [23,24]. Furthermore, MCC950, an NLRP3 inflammasome inhibitor, ameliorated podocyte injury, renal fibrosis, and renal dysfunction in db/db mice [25], and gasdermin D (GSDMD) knockdown using small interfering RNA (siRNA) in podocytes reversed hyperglycemia-induced inflammation and cell death [26]. Moreover, podocyte-specific gain- or loss-of-function studies have recently revealed that podocyte-specific NLRP3 inflammasome activation can promote DN [27]. Although these reports reveal an important role for NLRP3 inflammasome activation in podocytes during DN progression, the involvement of NLRP3 inflammasome activation in LPA-induced podocyte injury remains unclear. Therefore, in the present study, we investigated the effects and mechanisms of blocking LPA/LAPR1 signaling in podocyte damage during DN.

## 2. Results

### 2.1. AM095 Inhibits Podocyte Loss and Death in the Kidney of STZ-Induced Diabetic Mice

**Figure 1:**
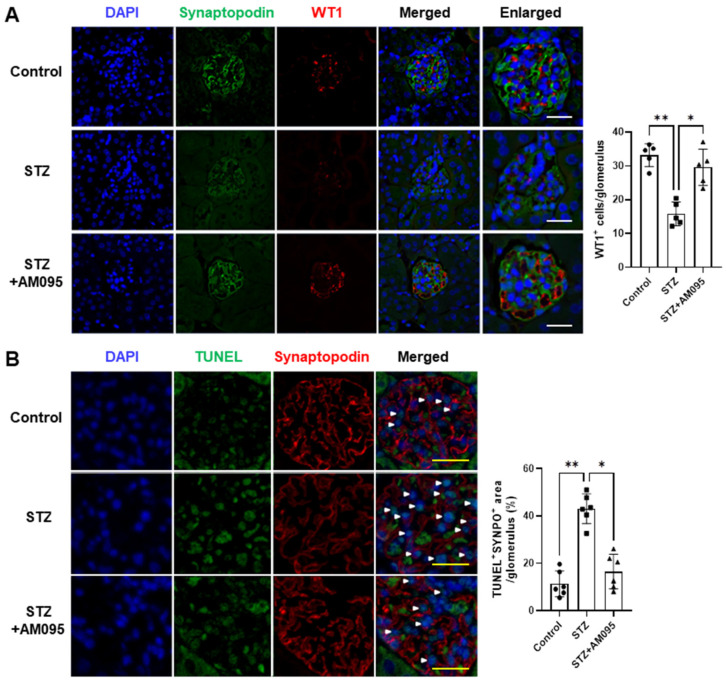
AM095 inhibits podocyte loss and death in the kidneys of STZ-induced diabetic mice. STZ-induced diabetic mice were orally administered vehicle (STZ) or 30 mg/kg AM095 (STZ + AM095) daily for 8 weeks and then sacrificed. Non-diabetic mice were administered equal amounts of the vehicle as a control (Control). (**A**) Representative immunofluorescence (IF) confocal images (left panel) of synaptopodin (green) and WT1 (red) in kidney tissue sections of mice. Nuclei were counterstained with DAPI (blue). Scale bars, 20 μm. Quantification graph (right panel) of WT1^+^ podocytes in the glomerulus is shown (*n* = 5 per group). (**B**) Representative IF confocal images (left panel) of TUNEL (green) and synaptopodin (red) in the glomeruli are shown. Nuclei were counterstained with DAPI (blue). The white arrowheads indicate the TUNEL^+^synaptopodin^+^ cells. Scale bars, 20 μm. Quantification graph (right panel) of apoptotic podocytes in the glomerulus is shown. The percentile of apoptotic podocytes was calculated as the TUNEL^+^synaptopodin^+^ area divided by the synaptopodin^+^ area in the glomerulus (*n* = 6 per group). Data are represented as the mean ± standard deviation (SD). *: *p* < 0.05, **: *p* < 0.01 via Kruskal–Wallis test with post hoc Dunn’s test for multiple comparisons.

Glomerular podocyte loss has been observed during DN progression in type 1 and type 2 diabetes [4,28]. We previously reported that STZ-induced type 1 diabetic mice exhibited weight loss, increased kidney weight, worsened renal function, upregulation of LPAR1 expression, and inflammation in the kidneys [13]. However, the administration of AM095, an LPAR1 inhibitor, attenuated the inflammatory response in STZ-induced diabetic mice, leading to a reduction in kidney weight, alleviation of albuminuria, and amelioration of glomerulosclerosis, thereby improving DN [13]. To determine whether AM095 administration can affect podocyte loss, we examined glomerular expression levels of podocyte markers, such as synaptopodin and WT1, in STZ-induced diabetic mice. The expression of podocyte markers was significantly reduced in the renal glomeruli of STZ-induced diabetic mice when compared with that in the control mice; however, AM095 administration could markedly restore this decrease in podocyte marker expression (Figure 1A). As apoptosis or cell death is considered a common mechanism of podocyte loss, we subjected kidney sections to TUNEL staining. As shown in Figure 1B, TUNEL^+^synaptopodin^+^ areas were significantly increased in the glomeruli of STZ-induced diabetic mice when compared with those of control mice; however, AM095 administration significantly decreased this cell population (Figure 1B). These results suggest that LPA/LPAR1 signaling could be associated with the progression of podocyte loss in DN.

### 2.2. AM095 Inhibits the Increased Expression of NLRP3 Inflammasome Factors in Podocytes of STZ-Induced Diabetic Mice

**Figure 2:**
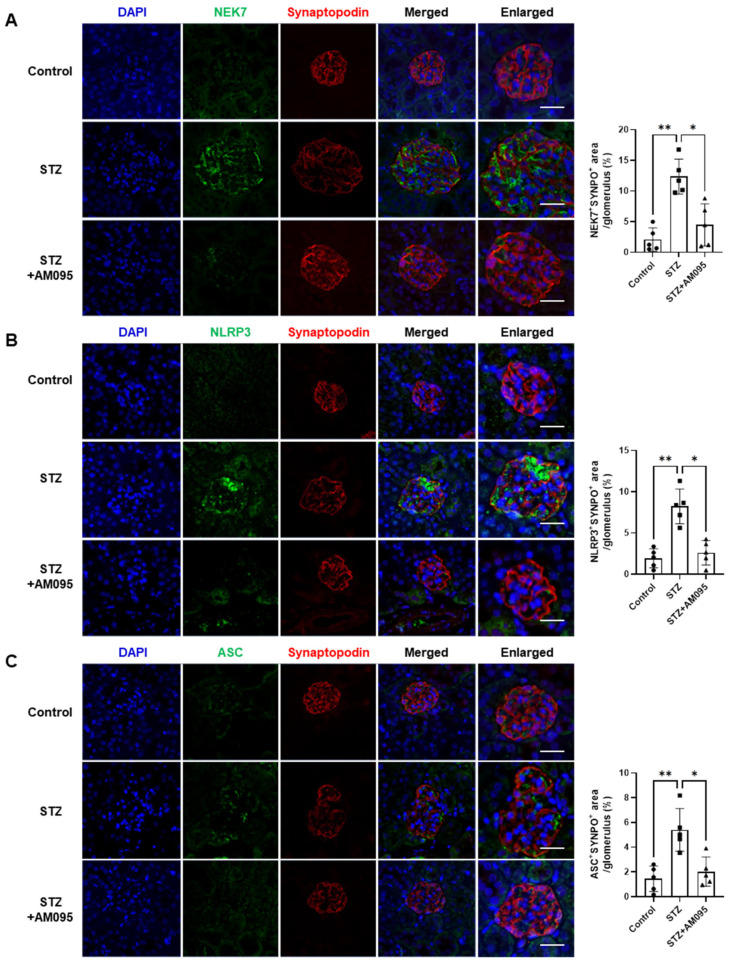
AM095 inhibits the increased expression of NLRP3 inflammasome factors in podocytes of STZ-induced diabetic mice. Representative immunofluorescence (IF) confocal images of (**A**) NEK7 (green), (**B**) NLRP3 (green), (**C**) ASC (green), and podocyte marker synaptopodin (red) in kidney tissue sections of mice. Nuclei were counterstained with DAPI (blue). Scale bars, 20 μm; *n* = 5 per group. Quantification graph (right panel) of each gene expression in synaptopodin^+^ area in the glomerulus is shown. Data are represented as the mean ± SD. *: *p* < 0.05, **: *p* < 0.01 via Kruskal–Wallis test with post hoc Dunn’s test for multiple comparisons.

NLRP3 inflammasome activation was observed in high-glucose-treated podocytes and diabetic db/db mice, and the inhibition of NLRP3 inflammasome activation could prevent podocyte damage in DN [25]. Given that AM095 administration inhibited podocyte loss in STZ-induced diabetic mice, we investigated the expression of NLRP3 inflammasome factors in podocytes in kidney sections. The expression levels of NEK7, NLRP3, and apoptosis-associated speck-like protein (ASC) in synaptopodin^+^ areas were significantly increased in STZ-induced diabetic mice when compared with control mice (Figure 2A–C). However, AM095 administration significantly suppressed the increased expression of these factors (Figure 2A–C). These results suggest that LPA/LPAR1 signaling participates in podocyte loss in DN via NLRP3 inflammasome activation.

### 2.3. AM095 Inhibits LPA-Induced Pyroptosis in E11 Cells

**Figure 3:**
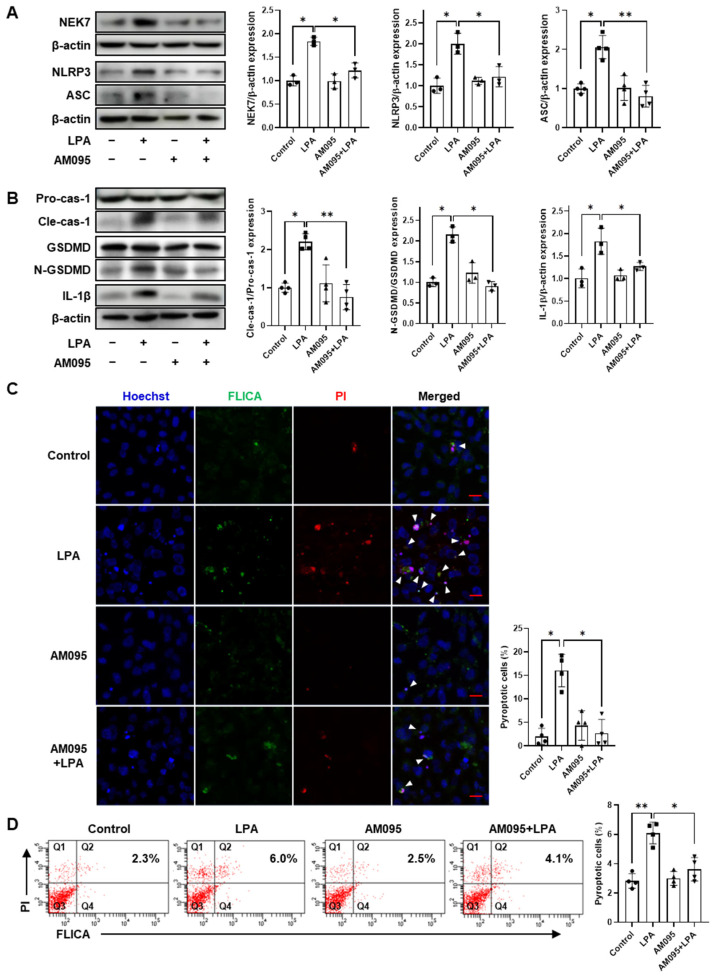
AM095 inhibits LPA-induced pyroptosis in E11 cells. (**A**,**B**) Differentiated E11 cells were treated with 5 µM LPA and 10 µM AM095 simultaneously for 3 (**A**) or 6 (**B**) h, or treated with either AM095 or LPA alone. Protein levels of NEK7, NLRP3, ASC (**A**), cleaved-caspase-1 (Cle-cas-1), N-GSDMD, and IL-1β (**B**) were analyzed via Western blotting, quantified using ImageJ software and normalized to those of β-actin, pro-caspase-1 (Pro-cas-1), and GSDMD, respectively (*n* = 3 or 4). (**C**,**D**) Differentiated E11 cells were treated with 5 µM LPA and 10 µM AM095 simultaneously for 4.5 h, or treated with either AM095 or LPA alone. (**C**) Cells were stained with FAM FLICA caspase-1 (green), propidium iodide (PI) (red), and Hoechst 33342 (blue). The white arrowheads indicate the pyroptotic cells (FLICA^+^PI^+^ cells). Scale bar: 20 μm. Representative images (left) and quantitative analysis scatter dot plot (right) are shown (*n* = 4). (**D**) Cells were stained with FAM FLICA caspase-1 and analyzed via flow cytometry immediately after adding PI. Representative flow cytometry scatter plots (left panel) and the percentage dotted plot of pyroptotic cells (Q2) (right panel) are shown (*n* = 4). Data are represented as the mean ± SD. *: *p* < 0.05, **: *p* < 0.01 via Kruskal–Wallis test followed by Dunn’s test for multiple comparisons and Mann–Whitney U test for two specific groups.

As we observed that AM095 administration alleviated podocyte loss and NLRP3 inflammasome factor expression in the kidneys of STZ-induced diabetic mice, we aimed to confirm these effects in E11 cells, a mouse podocyte cell line. LPA treatment significantly increased the expression of NLRP3 inflammasome factors, including NEK7, NLRP3, and ASC, in E11 cells; AM095 treatment significantly suppressed their LPA-induced expression (Figure 3A). NLRP3 inflammasome activation activates caspase-1, which cleaves GSDMD and pro-IL-1β to generate N-terminal GSDMD (N-GSDMD) and mature IL-1β, respectively [29]. LPA treatment significantly increased the expression of cleaved-caspase-1, N-GSDMD, and IL-1β when compared with control cells; however, AM095 treatment significantly decreased these LPA-induced expression levels (Figure 3B). To confirm whether LPA could induce pyroptosis in podocytes, we stained LPA-treated cells with FAM FLICA reagent for active caspase-1 and PI for cell death. LPA significantly increased the number of pyroptotic cells (FLICA^+^PI^+^ cells), but AM095 significantly suppressed this increased number (Figure 3C,D). These results suggest that LPA/LPAR1 signaling could induce pyroptosis in podocytes via NLRP3 inflammasome activation.

### 2.4. Egr1 Is Required for LPA-Induced Pyroptosis in E11 Cells

**Figure 4:**
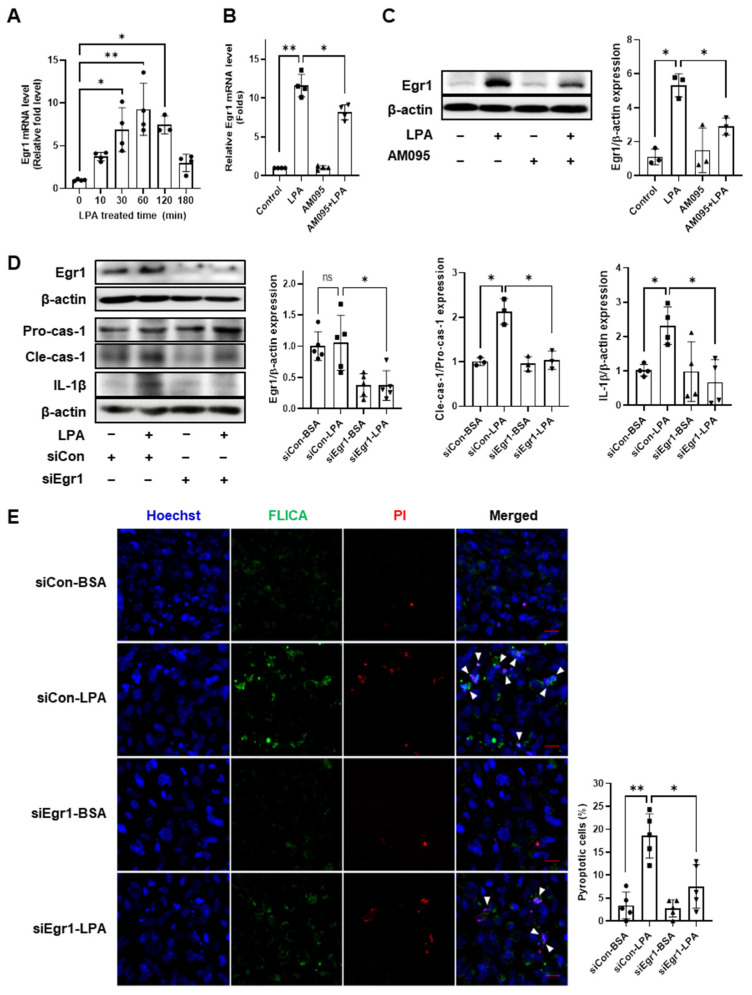
Egr1 is required for LPA-induced pyroptosis in E11 cells. (**A**) Differentiated E11 cells were treated with 5 µM LPA for 10, 30, 60, 120, and 180 min. (**B**,**C**) Cells were treated with 5 µM LPA and 10 µM AM095 simultaneously for 1 h, or with either AM095 or LPA alone. (**A**,**B**) Egr1 mRNA level was determined via RT-qPCR. (*n* = 4) (**C**) The Egr1 protein level was analyzed via Western blotting, quantified using the ImageJ software, and normalized to that of β-actin (*n* = 3). (**D**,**E**) Differentiated E11 cells were transfected with scrambled siRNA (siCon) or Egr1 siRNA (siEgr1) for 6 h, starved for 18 h, and then treated with 5 µM LPA for 4.5 (**E**) or 6 (**D**) h. (**D**) Protein levels of Egr1, cleaved-caspase-1 (Cle-cas-1), and IL-1β were measured using Western blotting, quantified using ImageJ software, and normalized to those of β-actin or pro-caspase-1 (Pro-cas-1), respectively (*n* = 3–5). (**E**) Cells were stained with FAM FLICA caspase-1 (green), propidium iodide (PI) (red), and Hoechst 33342 (blue). The white arrowheads indicate pyroptotic cells (FLICA^+^PI^+^ cells). Scale bar: 20 μm. Representative images (left) and quantitative analysis scatter dot plot (right) are shown (*n* = 5). Data are represented as the mean ± SD. ns, statistically non-significant. *: *p* < 0.05, **: *p* < 0.01 via Kruskal–Wallis test followed by Dunn’s test for multiple comparisons and Mann–Whitney U test for two specific groups.

The expression of transcription factor Egr1 (early growth response protein 1) has been reported to be increased in the renal cortex of diabetic mice [30] and regulates NLRP3 inflammasome activation in the podocytes of a S-adenosylhomocysteine hydrolase (SAHH) inhibition-induced DN model [20]. Given that the function of Egr1 in LPA-induced podocyte pyrolysis remains unknown, we first analyzed the expression levels of Egr1 in LPA-treated E11 cells. The mRNA levels of Egr1 were significantly increased in E11 cells 10 min after LPA treatment, reaching a maximum at 1 h, decreasing thereafter (Figure 4A). LPA-induced Egr1 mRNA and protein levels were significantly increased; however, AM095 treatment significantly decreased these increased levels (Figure 4B,C). To examine the role of Egr1 in LPA-induced pyroptotic factor expression and pyroptosis, we transfected E11 cells with Egr1 siRNA and cultured them in the presence or absence of LPA. We confirmed the knockdown of Egr1 expression by Egr1 siRNA transfection, and Egr1 knockdown significantly decreased the LPA-induced expression of cleaved-caspase-1 and IL-1β compared with that in scrambled siRNA-transfected cells (Figure 4D). Consistently, Egr1 knockdown markedly suppressed LPA-induced pyroptosis when compared with scrambled siRNA-transfected cells (Figure 4E). These results suggest that LPA-induced pyroptosis could be mediated by upregulated Egr1 expression in E11 cells.

### 2.5. Egr1 Knockdown Decreases LPA-Induced Nuclear Factor-κB (NF-κB) Activation and Desmin Expression in E11 Cells

**Figure 5:**
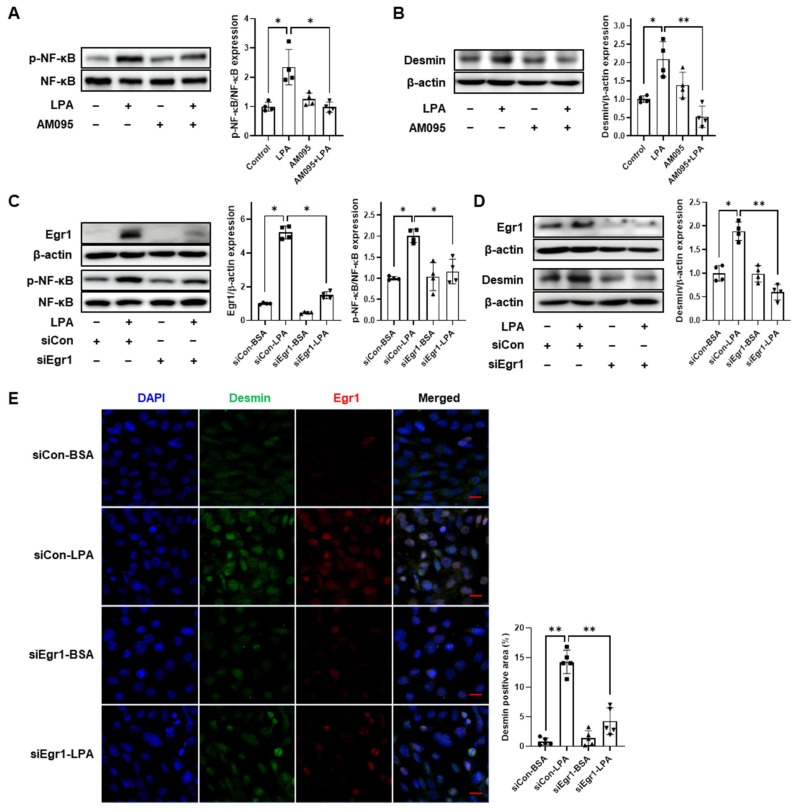
Egr1 knockdown decreases LPA-induced NF-κB activation and desmin expression in E11 cells. (**A**,**B**) Differentiated E11 cells were treated with 5 µM LPA and 10 µM AM095 simultaneously for 1 (for p-NF-κB, (**A**)) or 6 (for desmin, (**B**)) h, or treated with either AM095 or LPA alone. (**C**–**E**) Differentiated E11 cells were transfected with siCon or siEgr1 for 6 h, starved for 18 h, and then treated with 5 µM LPA for 1 (**C**) or 6 (**D**,**E**) h. Protein levels of p-NF-κB, Egr1, and desmin were analyzed by Western blotting, quantified using ImageJ software, and normalized to those of NF-κB or β-actin, respectively (*n* = 4). (**E**) Cells were stained with desmin (green), Egr1 (red), and DAPI (blue). Scale bar: 20 μm. Representative images (left) and quantitative analysis scatter dot plot (right) are shown (*n* = 5). Data are represented as the mean ± SD. *: *p* < 0.05, **: *p* < 0.01 via Kruskal–Wallis test followed by Dunn’s test for multiple comparisons and Mann–Whitney U test for two specific groups.

LPA induces NF-κB activation and inflammatory responses in various cells, including renal mesangial cells [13], Swiss 3T3 fibroblasts [31], and bronchial epithelial cells [32]. As NF-κB signaling is known to mediate NLRP3 inflammasome activation [33], we investigated the activation of NF-κB mediated by LPA/LPAR1 signaling in E11 podocytes. We found that LPA significantly increased NF-κB phosphorylation, whereas AM095 treatment significantly inhibited LPA-induced NF-κB activation (Figure 5A). Furthermore, NLRP3 inflammasome activation results in podocyte damage. Therefore, we examined the expression of desmin, a sensitive marker of podocyte damage, in E11 cells. LPA significantly increased desmin expression; however, AM095 treatment significantly suppressed desmin induction (Figure 5B). Given that Egr1 mediates LPA-induced NLRP3 inflammasome activation and pyroptosis in podocytes, we aimed to confirm whether Egr1 could also affect LPA-induced NF-κB activation and desmin expression in E11 cells. Egr1 siRNA transfection significantly inhibited LPA-induced Egr1 expression, and Egr1 knockdown significantly inhibited LPA-induced NF-κB phosphorylation when compared with that in scrambled siRNA-transfected cells (Figure 5C). Egr1 knockdown also significantly reduced LPA-induced desmin expression (Figure 5D,E). These results suggest that Egr1 could mediate the initiation of LPA-induced pyroptosis and podocyte damage.

### 2.6. LPA Increased Egr1 Expression by Downregulation of EzH2 in E11 Cells

**Figure 6:**
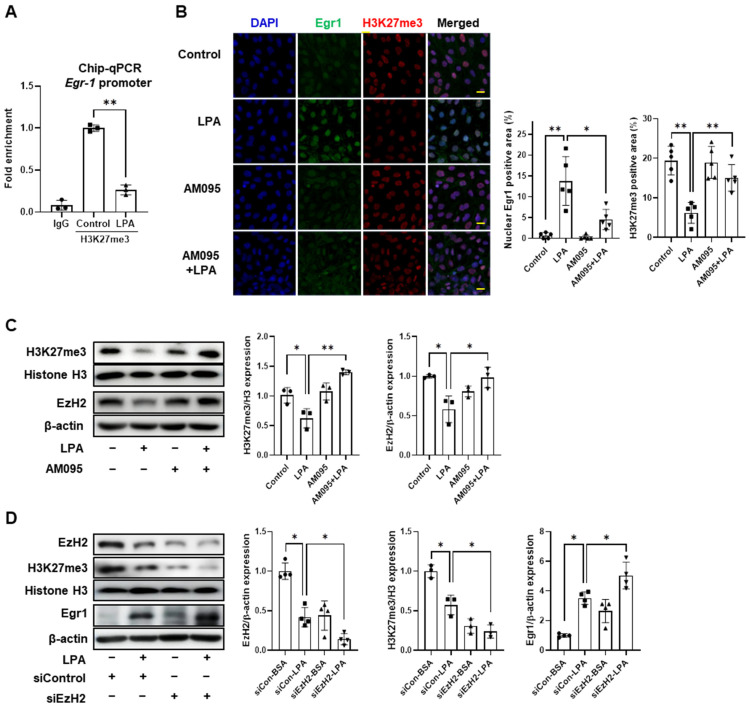
LPA increased Egr1expression by downregulation of EzH2 in E11 cells. (**A**) Differentiated E11 cells were treated with 5 µM LPA for 1 h, and chromatin immunoprecipitation (ChIP)-qPCR assay was performed to evaluate the enrichment of H3K27me3 at the Egr1 promoter region. A normal IgG antibody was used as a negative control (*n* = 3). (**B**,**C**) Differentiated E11 cells were treated with a combination of 5 µM LPA and 10 µM AM095 for 1 h, or treated with either AM095 or LPA alone. (**B**) Cells were stained with Egr1 (green), H3K27me3 (red), and DAPI (blue). Scale bar: 20 μm. Representative images (left) and quantitative analysis scatter dot plot (right) are shown (*n* = 5). (**D**) Differentiated E11 cells were transfected with siCon or EzH2 siRNA (siEzH2) for 6 h, starved for 18 h, and then treated with 5 µM LPA for 1 h. (**C**,**D**) Protein levels of H3K27me3, EzH2, and Egr1 were analyzed via Western blotting, quantified using ImageJ software, and normalized to those of Histone H3 (H3) or β-actin, respectively (*n* = 3 or 4). Data are represented as the mean ± SD. *: *p* < 0.05, **: *p* < 0.01 via Kruskal–Wallis test followed by Dunn’s test for multiple comparisons and Mann–Whitney U test for two specific groups.

It has been reported that high-glucose- and adenosine-dialdehyde-induced Egr1 expression in podocytes is mediated by a decrease in histone H3 lysine 27 trimethylation (H3K27me3) [23]. Therefore, we examined using a ChIP assay whether the LPA-induced Egr1 increase can be attributed to the same epigenetic regulation. We found that H3K27me3 enrichment at the promoter of the Egr1 gene was significantly reduced in LPA-treated E11 cells when compared with control cells (Figure 6A). Immunofluorescence staining confirmed that LPA increased nuclear Egr1 expression and decreased H3K27me3 expression; however, AM095 treatment suppressed these LPA-induced changes (Figure 6B). We further verified that the protein expression of H3K27me3 was significantly reduced in LPA-treated E11 cells when compared with control cells (Figure 6C). Furthermore, LPA significantly decreased the expression of histone methyltransferase EzH2 (Enhancer of Zeste Homolog 2), the enzyme responsible for methylating H3K27, in E11 cells (Figure 6C); however, AM095 treatment significantly inhibited this LPA-mediated effect (Figure 6C). To determine whether LPA-induced Egr1 expression was mediated by downregulating EzH2 expression, we transfected E11 cells with EzH2 siRNA, followed by LPA treatment. The knockdown of EzH2 was confirmed in siEzH2-transfected cells, and its expression was further decreased upon LPA treatment (Figure 6D). Consistently, EzH2 knockdown significantly reduced the LPA-mediated expression of H3K27me3 when compared with scrambled siRNA-transfected cells (Figure 6D). Conversely, EzH2 knockdown significantly increased LPA-induced Egr1 expression (Figure 6D). These results suggest that LPA-induced Egr1 expression is mediated by the downregulation of EzH2 and consequent reduction of H3K27me3.

### 2.7. AM095 Decreases Egr1 Expression and Restores EzH2 and H3K27me3 Expression in Podocytes from STZ-Induced Diabetic Mice

**Figure 7:**
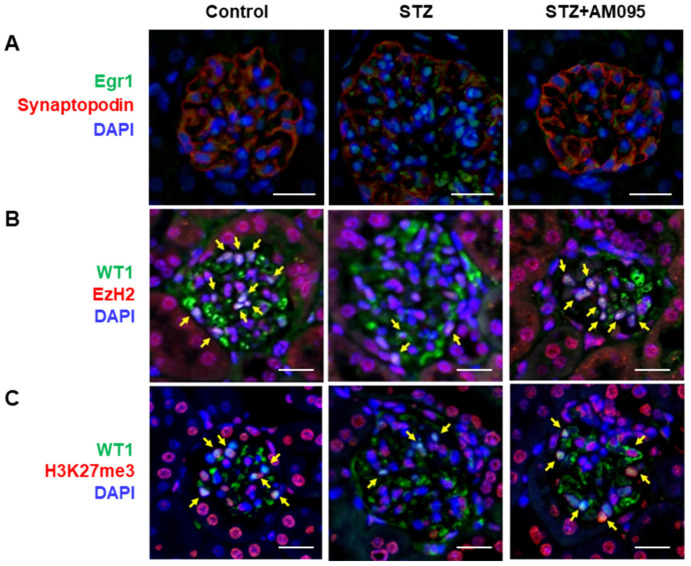
AM095 decreases Egr1 expression and restores EzH2 and H3K27me3 expression in podocytes from STZ-induced diabetic mice. Representative immunofluorescence (IF) confocal images of (**A**) Egr1 (green) and synaptopodin (red), (**B**) EzH2 (red), or (**C**) H3K27me3 (red), and podocyte marker WT1 (green) in kidney tissue sections of mice. Nuclei were counterstained with DAPI (blue). The yellow arrows indicate co-expressed podocytes. Scale bars, 20 μm; *n* = 3 per group.

Given that LPA-induced downregulation of EzH2 increased Egr1 expression in E11 cells, we aimed to verify these findings using an in vivo model. Similar to LPA-treated E11 cells, Egr1 expression was increased in podocytes, especially in the nuclei, from STZ-induced diabetic mice, which was suppressed by AM095 administration (Figure 7A). However, the expression levels of EzH2 and H3K27me3 were decreased in the podocytes of STZ-induced diabetic mice when compared with those in control mice; these levels were restored by AM095 administration (Figure 7B,C). Collectively, these results suggest that the downregulated expression of EzH2 and H3K27me3 participates in LPA-induced pyroptosis in podocytes by upregulating Egr1 expression.

## 3. Discussion

Previously, we revealed that AM095, an LPAR1 inhibitor, can ameliorate DN by suppressing TLR4/NF-κB signaling-mediated inflammation in the mesangial cells of STZ-induced diabetic mice [13]. Herein, we investigated the role of LPAR1 signaling in podocyte damage and found that inhibiting LPA/LPAR1 signaling could alleviate podocyte damage by suppressing NLRP3 inflammasome activation in the podocytes of STZ-induced diabetic mice. In vitro, LPA/LPAR1 signaling decreased H3K27 histone trimethylation at the Egr1 promoter by downregulating EzH2, increasing Egr1 expression, thereby leading to NLRP3 inflammasome activation, pyroptosis, and podocyte damage.

Glomerular podocytes are highly differentiated cells with complex cytoarchitecture. Hyperglycemia can change podocyte phenotype by inducing the loss of nephrin, altering the production/degradation of extracellular matrix components, and enhancing pro-trophic cytokine-modifying growth factors [28]. However, LPA has been recently identified as another important factor in DN pathogenesis [12,13,14,15]. Zhang et al. reported that LPAR1/3 inhibition in type 2 diabetic mice restored podocyte marker expression and the podocyte number in glomeruli [15]; however, the underlying mechanisms remain unclear.

Similar to the type 2 DN model (eNOS^−/−^ db/db mice) [15], the expression of podocyte markers, including synaptopodin and WT1, was significantly reduced in the glomeruli of STZ-induced diabetic mice (Figure 1A). Compared with control mice, eNOS^−/−^ db/db mice exhibit significantly increased LPAR1 and LPAR3 expression in the kidney, co-expressed with podocyte markers [15]. However, STZ-induced diabetic mice demonstrate a significant increase in LPAR1 mRNA and protein expression in the kidney [13]. Although a recent cloud-based tool has been reported for accurate identification of podocytes, the current gold standard for podocyte counting in immunostainings is the use of WT1, a podocyte-specific nuclear marker [34]. In this study, the expression of nuclear WT1 and cytoplasmic synaptopodin were confirmed as markers for podocyte identification. Except for WT1, synaptopodin staining had certain limitations during podocyte counting; however, the expression patterns of both markers were found to be similar within each group (Figure 1A). Moreover, despite the reduced intensity of synaptopodin staining in STZ-induced diabetic mice, an increase in TUNEL^+^ cells was observed. However, AM095-mediated LPAR1 inhibition restored podocyte marker expression and reduced TUNEL^+^synaptopodin^+^ area in STZ-induced diabetic mice (Figure 1). Accordingly, LPA/LPAR1 signaling could contribute to podocyte loss in DN pathogenesis.

NLRP3 inflammasome activation reportedly mediates podocyte damage in db/db type 2 diabetic mice and STZ-induced type 1 diabetic mice [24,25]. However, diabetes-induced podocyte loss and glomerular lipid accumulation were suppressed in STZ-injected NLRP3 knockout mice or NLRP3 inflammasome-specific inhibitor-administered db/db mice [25]. Shahzad et al. reported that podocyte-specific NLPR3 inflammasome activation promotes DN [27], indicating the importance of NLRP3 inflammasome activation in podocyte injury during DN progression. We examined the expression of NLRP3 inflammasome factors in the podocytes of STZ-induced diabetic mice administered AM095. AM095 significantly inhibited the expression of NLRP3 and ASC, as well as NEK7, which is important for regulating NLRP3 inflammasome activation [35,36] (Figure 2).

Using differentiated E11 cells, we confirmed that LPA induced NLRP3 inflammasome activation in podocytes in vitro. Similarly to in vivo results, LPA markedly increased expression levels of NLRP3, ASC, and NEK7 in E11 cells (Figure 3). In our preliminary study, we detected LPAR1 and LPAR2, but not LPAR3, in differentiated E11 podocytes, with only LPAR1 expression increased upon high glucose exposure (Supplementary Figure S1). Accordingly, we employed AM095 and found that it could significantly suppress the LPA-induced expression of NLRP3 inflammasome factors in E11 cells (Figure 3). Upon NLRP3 inflammasome activation, cleaved-caspase-1 cleaves cytokine precursors pro-IL-1β and pro-IL-18 to release mature IL-1β and IL-18 [37,38] and promotes pyroptosis, a form of inflammatory cell death, which is regulated by pore formation in the plasma membrane by N-GSDMD [38,39]. Next, we investigated whether LPA induced NLRP3 inflammasome-mediated pyroptosis in E11 cells. LPA significantly enhanced the expression of pyroptotic factors, consequently increasing pyroptosis; however, AM095 treatment significantly suppressed LPA-induced expression of pyroptotic factors and pyroptosis (Figure 3). Both in vivo and in vitro results indicate that LPA induced NLRP3 inflammasome activation and pyroptosis in podocytes via LPAR1.

In DN, Egr1 reportedly mediates cell proliferation and fibrosis induced by high glucose or LPA in glomerular mesangial cells [40,41]. Egr1 is a zinc-finger transcription factor of the immediate early response family, highly expressed in the kidneys of diabetic rats and mice [30,41]. Egr1 participates in thioredoxin-interacting protein (TXNIP)-mediated oxidative stress and NLRP3 inflammasome activation in the podocytes of SAHH inhibition-aggravated DN [23]. Therefore, we hypothesized that Egr1 might play a role in LPA-induced NLRP3 inflammasome activation in podocytes. We found that LPA induced Egr1 expression through LPAR1 and Egr1 knockdown inhibited both LPA-induced expression of pyroptotic factors, including cleaved-caspase-1 and IL-1β, and pyroptosis in E11 cells (Figure 4). Moreover, Egr1 deficiency can attenuate NLPR3 inflammasome activation and renal inflammation in a mouse model of adenine-induced renal injury [42]. We observed that Egr1 knockdown significantly inhibited LPA-induced NF-κB phosphorylation (Figure 5). NF-κB is a crucial mediator of NLRP3 inflammasome activation [33]. Pang et al. detected a physical interaction between Egr1 and NF-κB p65 and reported that Egr1 deficiency reduced NF-κB activation in *Pseudomonas aeruginosa*-infected macrophages [43]. Similarly, Egr1 deficiency lowers NF-κB activity by reducing the level of IKKα/β phosphorylation in the kidneys in adenine-induced renal injury [42]. These results suggest the involvement of Egr1 in LPA-induced NF-κB activity by directly interacting with NF-κB or via the regulation of upstream factors. Ho et al. reported that cytoplasmic and nuclear expression levels of Egr1 were significantly increased in the kidneys of patients with diabetic renal failure when compared with those in patients with normal kidneys [42]. Accordingly, Egr1 may participate in LPA-induced NLRP3 inflammasome activation and pyroptosis in DN.

Desmin, an intermediate filament protein, is a widely employed podocyte damage marker, and its expression was increased in the renal glomerular podocytes of diabetic mice and high-glucose-treated podocytes [44,45]. Therefore, we examined LPA-induced desmin expression in E11 cells and found that LPA/LPAR1 signaling induced desmin expression (Figure 5). Desmin expression has been positively correlated with NLRP3 inflammasome activation in primary glomerulonephritis [46]. This is consistent with the fact that LPA increases both desmin expression and NLRP3 inflammasome activation; however, further studies are needed to confirm a direct role of LPA between them. We also found that Egr1 knockdown significantly suppressed LPA-induced desmin expression in E11 cells. Zhang et al. measured desmin expression as a cell growth and differentiation marker in skeletal muscle satellite cells, reporting that Egr1 knockdown and overexpression could inhibit and increase desmin expression, respectively [47]. These results suggest that Egr1 can regulate desmin expression.

In addition, increased expression of TGF-β is a considerable feature of the pathogenesis of diabetic renal diseases, including podocyte injury [48]. The genetic overexpression of TGF-β in mice induces renal fibrosis through the upregulation of Egr1, STAT3, and AP-1 [49]. Interactions between TGF-β and Egr1 have been reported in various renal cell types. High glucose treatment activated the TGF-β1/Smad pathway mediated by Egr1 in HK-2 cells [50], and it increased Egr-1 expression in podocytes through the TGF-β1-ERK1/2 pathway [51]. Egr1 overexpression in mesangial cells induced TGF-β expression, whereas Egr1 knockdown suppressed TGF-β expression mediated by the diabetes-associated long non-coding RNA NONHSAG053901 [52]. In addition, a considerable increase in TGF-β1 mRNA expression in the kidneys of STZ-induced diabetic mice was observed, which was suppressed by administration of AM095 [13]. Moreover, LPA treatment increased TGF-β1 mRNA expression in mesangial cells, which was inhibited by AM095 [13]. In the present study, we observed that LPA increased TGF-β expression in E11 podocytes, which was inhibited by AM095 (Supplementary Figure S2). Furthermore, Egr1 knockdown reduced LPA-induced TGF-β expression in E11 podocytes (Supplementary Figure S2). These findings suggest that Egr1 plays a regulatory role in inducing TGF-β expression in renal cells in response to factors such as LPA and high glucose levels during DN progression.

Subsequently, we investigated the molecular mechanism underlying LPA-induced Egr1 expression in E11 cells. The epigenetic regulation of the expression of NLRP3 inflammasome components and transcription factors that target them has been suggested [53]. Epigenetic regulation, including histone modification, participates in chromatin remodeling and the regulation of gene expression [54]. Histone modifications include mainly methylation, acetylation, and phosphorylation [55]. Histone methylation can modulate chromatin structure; therefore, methylation can induce transcriptional silencing and activation [54]. In particular, lysine can undergo mono-, di-, and tri-methylation, and H3K27me3 is associated with transcriptional repression [56,57]. High glucose and adenosine dialdehyde treatment induced Egr1 expression by decreasing H3K27me3 enrichment at the Egr1 promoter in podocytes and induced TXNIP-mediated NLRP3 inflammasome activation [23]. We found that LPA reduced H3K27me3 enrichment at the Egr1 promoter in E11 cells. Consistently, LPA reduced the expression of the methyltransferase EzH2, which methylates H3K27, through LPAR1. Likewise, diabetic factor AGEs can reduce EzH2 expression in podocytes, consequently reducing H3K27me3 expression and increasing the expression of TGFβ1, a pathological factor contributing to podocyte damage [58]. In addition, EzH2 depletion in diabetic conditions exacerbates podocyte damage [59]. We also found that EzH2 knockdown in E11 cells further increased LPA-induced Egr1 expression. These results indicate that LPA/LPAR1 signaling downregulates EzH2 in podocytes, which decreases H3K27me3 enrichment at the Egr1 promoter and consequently increases Egr1 expression.

Furthermore, the LPA-mediated altered expression of factors such as Egr1, EzH2, and H3K27me3 in E11 cells was consistently observed in podocytes of STZ-induced diabetic mice (Figure 7). Renal H3K27me3 levels were reduced in OVE26 type 1 diabetic mice, inducing the mRNA expression of DN-related genes, including cyclooxygenase-2, fibroblast-specific protein 1, and monocyte chemoattractant protein-1 [60]. Reduced EzH2 expression and a corresponding decrease in H3K27me3 were observed in podocytes from db/db type 2 diabetic mice, which was confirmed to mediate podocyte damage [58]. Furthermore, the reduced expression of the EzH2/H3K27me3 axis could mediate the induction of podocyte damage in an adriamycin nephrotoxicity mouse model [61], and pharmacological inhibition of EzH2 could induce podocyte damage in mice and rats [59]. Accordingly, podocyte damage can be induced by EzH2/H3K27me3 reduction, and LPA/LPAR1 signaling may be one of several factors that induce podocyte damage.

EzH2 expression is mainly regulated by various transcription factors, including Myc and E2F, microRNAs (miRNAs) such as miR-26, miR-101, miR-21, and miR-214, or ubiquitination-mediated protein degradation [62,63]. miRNAs are small non-coding single-stranded RNAs that regulate expression levels of target gene mRNA via degradation or inhibition of translation by RNA interference [64]. Various miRNAs, including miR-21, miR-27a, and miR-29a, participate in the inflammatory response and podocyte death in DN [65]. In addition, LPA/LPAR1 signaling in basal breast cancers increases the expression of various miRNAs, including miR-21 [66]. These results suggest that podocytes could down-regulate EzH2 expression via LPA-induced increase in miRNAs. Conversely, EzH2 ubiquitination is critical for protein stability, and Smurf2, β-TrCP, and PRAJA1 function as E3 ligases to ubiquitinate EzH2 and promote its degradation [62]. Further studies are needed to elucidate the mechanism underlying LPA-induced EzH2 downregulation.

**Figure 8:**
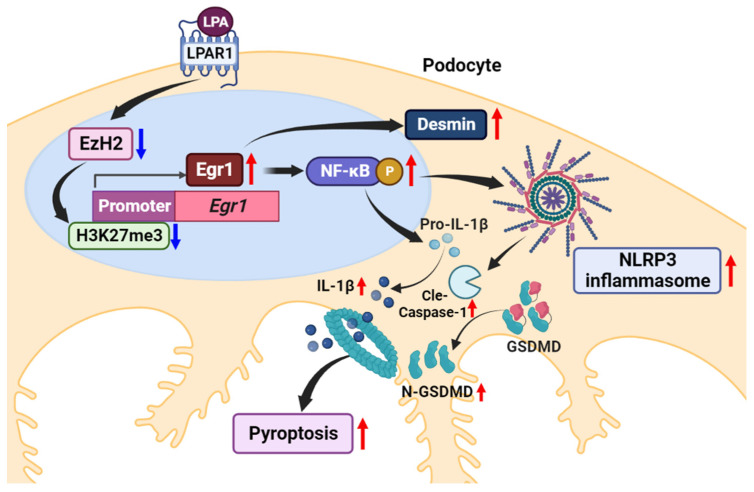
Schematic representation of the mechanism through which LPA/LPAR1 signaling in renal podocytes induces Egr1 expression via downregulation of EzH2/H3K27me3 expression, which induces NLRP3 inflammasome activation and pyroptosis. Created with BioRender.com. Red and blue arrows indicate upregulated and downregulated responses, respectively.

Taken together, our results show that LPA induces the expression of Egr1 through LPAR1, which activates the NLRP3 inflammasome and induces podocyte damage and death in the progression of DN (Figure 8). In particular, we demonstrated that the downregulation of EzH2 by LPA and resulting reduced enrichment of H3K27me3 in the Egr1 promoter contributes to the induction of Egr1 expression. Our findings may be one of the underlying mechanisms of LPA-induced damage in podocytes during DN progression and may provide a potential novel target for the treatment of DN.

## 4. Materials and Methods

### 4.1. Animal Studies

Male C57BL/6J mice (8 weeks old) were obtained from Orient Bio, Inc. (Seongnam, Gyeonggi-do, Republic of Korea). Mice were allowed free access to food (standard chow diet) and water and were housed in groups of 3–5 animals/cage under a 12 h light/dark cycle at room temperature (24 ± 2 °C), with constant humidity (40–50%). After adaptation for one week, type 1 diabetes was induced by administering an STZ injection, except in the normal control group. Mice were administered a daily intraperitoneal (i.p.) injection of 50 mg/kg/day STZ (S0130; Sigma-Aldrich, St. Louis, MO, USA) after fasting for 4 h in the morning for five consecutive days. Diabetes was confirmed by a blood glucose level >350 mg/dL. STZ-induced diabetic mice with similar body weights were randomly assigned to STZ+vehicle (STZ; *n* = 10) and STZ + AM095 (*n* = 10) treatment groups. AM095 (30 mg/kg; Sigma-Aldrich) or vehicle was administered orally via a gastric cannula once daily in the morning for 8 weeks. Age-matched C57BL/6J male mice were used as the non-diabetic control (control; *n* = 10) group. The animals were euthanized using carbon dioxide (CO_2_) gas inhalation according to established protocols. All animal experiments were conducted in accordance with a protocol approved by the Institutional Animal Care and Use Committee of the Lee Gil Ya Cancer and Diabetes Institute, Gachon University (Incheon, Republic of Korea; LCDI-2018-0115) [13].

### 4.2. Immunohistochemistry (IHC) and Immunocytochemistry

The right kidney with the renal capsule removed was fixed in formalin, embedded in a paraffin block, and sliced into 4 µm-thick sections. E11 cells (5 × 10^3^ cells/well) were seeded in a Nunc Lab-Tek II 4-well glass chamber (Thermo Fisher Scientific, Waltham, MA, USA) and allowed to differentiate for 14 days. After starvation, the cells were treated with 5 μM LPA and 10 μM AM095 simultaneously for 1 h or with either AM095 or LPA alone to evaluate the expression levels of Egr1 and H3K27me3. In addition, E11 cells transfected with siRNA were either treated with 5 μM LPA for 6 h or left unstimulated to evaluate desmin expression. Kidney sections or cell slides were stained as described previously [67], with the primary antibodies described in Table 1. Subsequently, kidney sections or cell slides were counterstained with 4,6-diamidino-2-phenylindole (DAPI; Invitrogen, Carlsbad, CA, USA) to detect nuclei and mounted with a fluorescence mounting medium (Dako North America, Inc., Carpinteria, CA, USA). The prepared samples were observed using a confocal microscope LSM 700 (Carl Zeiss Inc., Oberkochen, Germany) at the Core-facility for Cell to In-vivo imaging. The quantification of fluorescence intensity was performed with ImageJ 1.51v software (National Institutes of Health (NIH), Bethesda, MD, USA). The stained images were converted to the RGB stack type (Image-Type-RGB stack), and the threshold was adjusted (Image-Adjust-Threshold) for analysis, with the measured values chosen as area ratio or count [68].

### 4.3. TUNEL Assay

The kidney sections were prepared and stained with synaptopodin (anti-SYNPO, PA5-56997; Thermo Fisher Scientific) as described in Section 4.2. TUNEL staining was used to examine apoptosis in mouse podocytes (synaptopodin^+^ cells) and was performed using a TUNEL apoptosis detection kit (Green Fluorescence) (KTA2010; Abbkine, Wuhan, China), in accordance with the manufacturer’s instructions. The sections were then counterstained with DAPI (Invitrogen) for detecting nuclei and mounted with a fluorescence mounting medium (Dako North America, Inc.). The sections were observed using a confocal microscope LSM 700 (Carl Zeiss Inc.) at Core-facility for Cell to In-vivo imaging. The percentile of apoptotic podocytes was calculated as the number of TUNEL^+^synaptopodin^+^ cells divided by the number of synaptopodin^+^ cells in the glomeruli.

### 4.4. Cell Culture and Treatment

The murine kidney podocyte cell line, E11, was obtained from Cell Lines Service (Eppelheim, Germany). Undifferentiated E11 cells were plated in flasks precoated with collagen type I (Corning, Corning, NY, USA) and maintained in RPMI-1640 medium (Welgene Inc., Daegu, Republic of Korea), supplemented with 10% fetal bovine serum (FBS; Life Technologies, Grand Island, NY, USA), 1% penicillin–streptomycin (Welgene Inc.), and 10 U/mL recombinant mouse interferon (IFN)-γ (Miltenyi Biotec, Auburn, CA, USA) at 33 °C in an atmosphere of 5% CO_2_ and 95% air. To induce differentiation, the E11 cells were maintained at 37 °C without IFN-γ for 14 days.

To evaluate the effects of LPA, the medium of differentiated E11 cells was replaced with serum-free medium (SFM) containing 0.1% fatty acid-free bovine serum albumin (Sigma-Aldrich) and incubated for 18 h. The cells were treated with 5 μM LPA (Avanti Polar Lipids, Birmingham, AL, USA) in the presence or absence of 10 μM AM095 (Sigma-Aldrich).

### 4.5. Transfection of E11 Podocytes

The medium of differentiated E11 cells was replaced with penicillin- and streptomycin-free culture medium and cultured for 48 h. The cells were transfected with siRNA against Egr1 or EzH2 or, alternatively, scrambled siRNA (control) using TransIT-TKO^®^ Transfection Reagent (Mirus, Madison, WI, USA) according to the manufacturer’s instructions. All siRNAs were purchased from Bioneer, Inc. (Daejeon, Republic of Korea). After 6 h, the medium was replaced with SFM, and cells were cultured for 18 h. Subsequently, the cells were treated with 5 μM LPA for 1 h for assessing H3K27me3 and phosphorylated NF-κB expression, 4.5 h for investigating pyroptosis, and 6 h for examining Egr1, cleaved-caspase-1 (Cle-cas-1), IL-1β, and desmin expression. Alternatively, cells were maintained in an unstimulated state.

### 4.6. Western Blot Analysis

Total cellular protein extraction and Western blotting were performed as previously described [69]. For each target protein, lysate samples with equal protein amounts (10–50 µg total) were incubated overnight at 4 °C with the primary antibodies listed in Table 1. The membranes were then incubated with horseradish peroxidase-conjugated secondary antibodies, goat anti-mouse IgG (Invitrogen), or goat anti-rabbit IgG (Jackson ImmunoResearch, West Grove, PA, USA). The blots were developed using Immobilon-Western chemiluminescent HRP substrate (Millipore Corp., Billerica, MA, USA) and visualized using the LAS4000 imaging system (Fujifilm Corp., Tokyo, Japan) or Amersham ImageQuant™ 800 systems (Cytiva Life Sciences, formerly GE Healthcare Life Sciences, MA, USA). The relative intensities of the protein bands were quantified using ImageJ 1.51v software (NIH).

### 4.7. Determination of Pyroptosis

The E11 cells were seeded in a Nunc Lab-Tek II 4-well glass chamber (5 × 10^3^ cells/well; Thermo Fisher Scientific) or 12-well culture plates (1 × 10^4^ cells/well; Corning, New York, NY, USA), followed by differentiation for 14 days. LPA-induced pyroptosis was evaluated using the FAM FLICA Pyroptosis/Caspase-1 Assay kit (#9146; ImmunoChemistry Technologies, Bloomington, MN, USA) according to the manufacturer’s instructions. Briefly, differentiated E11 cells were treated with a combination of 5 μM LPA and 10 μM AM095 or individually with AM095 or LPA for 4.5 h. Similarly, siRNA-transfected cells were treated with 5 μM LPA for 4.5 h or maintained without stimulation. After 4.5 h of incubation, FAM FLICA reagent was added to the cells to measure the expression level of active caspase 1. After additional culturing, the cells were washed and stained with propidium iodide (PI; Sigma-Aldrich). The nuclei were counterstained with Hoechst 33342. Subsequently, the cells were mounted with a fluorescence mounting medium (Dako North America, Inc.) and observed under a confocal microscope LSM 700 (Carl Zeiss Inc.) at the Core-facility for Cell to In-vivo imaging. For flow cytometry, the LPA-treated cells were harvested and stained with FAM FLICA, and PI was added immediately before analysis. The stained cells were analyzed via flow cytometry in a FACS LSRII using CellQuest™ Pro Software version 7.0 (BD Biosciences, San Jose, CA, USA) at the Core-facility for Cell to In-vivo imaging.

### 4.8. Reverse Transcription-Quantitative PCR (RT-qPCR) Analysis

The total RNA was extracted from cells using RNAiso Plus reagent (Takara Bio Inc., Kyoto, Japan) according to the manufacturer’s instructions. The cDNA was synthesized using the PrimeScript First Strand cDNA Synthesis Kit (Takara Bio Inc.). RT-qPCR was performed using a reaction mixture of SYBR Premix Ex Taq II (Takara Bio Inc.) in a CFX384TM Real-Time PCR System (Bio-Rad, Hercules, CA, USA). The Egr1 primer sequences were 5′-CACTCACCCACCATGGACAA-3′ and 5′-CCCGTTGCTCAGCAGCAT-3′. The 18s rRNA was used as an internal control, with the following primer sequences: 5′-CCATCCAATCGGTAGTAGCG-3′ and 5′-GTAACCCGTTGAACCCCATT-3′. The relative gene expression levels were normalized to the expression levels of 18s rRNA and calculated using the 2^−ΔΔCT^ relative quantification method.

### 4.9. Chromatin Immunoprecipitation (ChIP)-qPCR Assay

The differentiated E11 cells were treated with or without LPA for 1 h. The ChIP assay was performed using a Pierce™ Magnetic ChIP Kit (#26157; Thermo Fisher Scientific) according to the manufacturer’s instructions. The immunoprecipitation reactions were performed using 1 μg of H3K27me3 (#9733; Cell Signaling Technology) or normal rabbit IgG (Thermo Fisher Scientific) antibodies. The primer sequences in the mouse Egr1 promoter used for ChIP-qPCR were forward, 5′-CCCGGGGGCCGTC-3′ and reverse, 5′-CCCAAATAAGGTCTGTTCCG-3′ [70].

### 4.10. Statistical Analysis

The statistical analyses were performed using GraphPad Prism version 9.5.1 (GraphPad Software Inc., San Diego, CA, USA). The data were analyzed using a non-parametric Kruskal–Wallis test. If there was a significant difference among groups, the post hoc Dunn’s test for multiple comparisons was performed. In some cases where the Kruskal-Wallis test indicated “*p* < 0.05 and Exact”, but no significance was observed in the post hoc Dunn’s multiple comparisons test, further comparison of specific two groups was performed using the non-parametric Mann–Whitney U test. All results are presented as mean ± standard deviation (SD). Statistical significance was set at *p* < 0.05.

## Figures and Tables

**Table 1:** List of antibodies.

Antibody	Company	Cat. No.	Dilution Ratio
β-actin (C4)	Santa Cruz Biotechnology (Dallas, TX, USA)	SC-47778	WB 1:3000
Egr-1 (C-19) (rabbit)	Santa Cruz Biotechnology (Dallas, TX, USA)	sc-189	WB 1:1000; IFA 1:200
Egr-1 (B-6)	Santa Cruz Biotechnology (Dallas, TX, USA)	sc-515830	IFA 1:200
Human ZIF268/EGR1 Antibody	LSBio (Lynnwood, WA, USA)	LS-C104940-100	IFA 1:400
Desmin (RD301)	Santa Cruz Biotechnology (Dallas, TX, USA)	sc-23879	WB 1:1000; IFA 1:200
Caspase-1	Enzo Life Sciences (Farmingdale, NY, USA)	ALX-210-804-C100	WB 1:1300
IL-1β	Santa Cruz Biotechnology (Dallas, TX, USA)	SC-52012	WB 1:1000
Ezh2 (D2C9)	CST (Danvers, MA, USA)	5246	WB 1:2000; IFA 1:200
Tri-Methyl-Histone H3 (Lys27) (C36B11) (rabbit)	CST (Danvers, MA, USA)	9733S	WB 1:2000; IFA 1:200
Histone H3 (D1H2) XP^®^ (rabbit)	CST (Danvers, MA, USA)	4499S	WB 1:2000
SYNPO Polyclonal Antibody	Invitrogen (Waltham, MA, USA)	PA5-56997	WB 1:1000; IFA 1:400
WT1(C-19)	Santa Cruz Biotechnology (Dallas, TX, USA)	sc-192	WB 1:500; IFA 1:100
NEK7 (A-12)	Santa Cruz Biotechnology (Dallas, TX, USA)	sc-398439	WB 1:1000; IFA 1:100
NLRP3/NALP3 Antibody—BSA Free (rabbit)	Novus Biologicals (Littleton, CO, USA)	NBP2-12446	WB 1:2000
Human/Mouse NLRP3/NALP3 Antibody (rat)	R&D system (Minnesota, MN, USA)	MAB7578	IFA 1:200
ASC (B-3)	Santa Cruz Biotechnology (Dallas, TX, USA)	sc-514414	WB 1:1000; IFA 1:100
GSDMDC1	Novus Biologicals (Littleton, CO, USA)	NBP2-33422	WB 1:1000
Phospho-NF-κB p65 (Ser536) (93H1) (rabbit)	CST (Danvers, MA, USA)	3033	WB 1:2000
NF-κB p65 (C22B4) (rabbit)	CST (Danvers, MA, USA)	4764	WB 1:2000

CST: Cell Signaling Technology; Invitrogen: Thermo Fisher Scientific; LSBio: LifeSpan BioSciences; WB: Western blotting; IFA: immunofluorescence assay.

## Data Availability

The data used to support the findings of this study are available from the corresponding author upon request.

## Supplementary Materials

The following supporting information can be downloaded at: https://www.mdpi.com/article/10.3390/ijms24129968/s1.
