# Supplementary material for: Lysophosphatidic Acid Induces Podocyte Pyroptosis in Diabetic Nephropathy by an Increase of Egr1 Expression via Downregulation of EzH2
Source: Int J Mol Sci. 2023 Jun 9;24(12):9968. doi: 10.3390/ijms24129968 (PMC10298531; doi:10.3390/ijms24129968)
Supplement: Supplementary file 1 [file ijms-24-09968-s001.zip › ijms-2383196-supplementary.pdf]

Supplementary Figure S1.

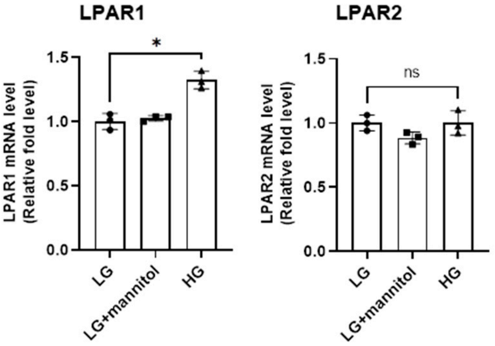

Figure S1. High glucose increases LPAR1 expression in E11 cells. Differentiated E11 cells were treated with low glucose (LG; 5 mM), LG (5 mM) + mannitol (25 mM), and high glucose (HG; 30 mM) for 24 h. mRNA levels of lysophosphatidic acid receptor (LPAR)1-5 were determined by RT-qPCR (*n* = 3). mRNA levels of LPAR3–5 were not detected. Data are represented as the mean ± standard deviation (SD). ns, statistically non-significant. *: $p < 0.05$ via Kruskal–Wallis test followed by Mann–Whitney U test for two specific groups.

Supplementary Figure S2.

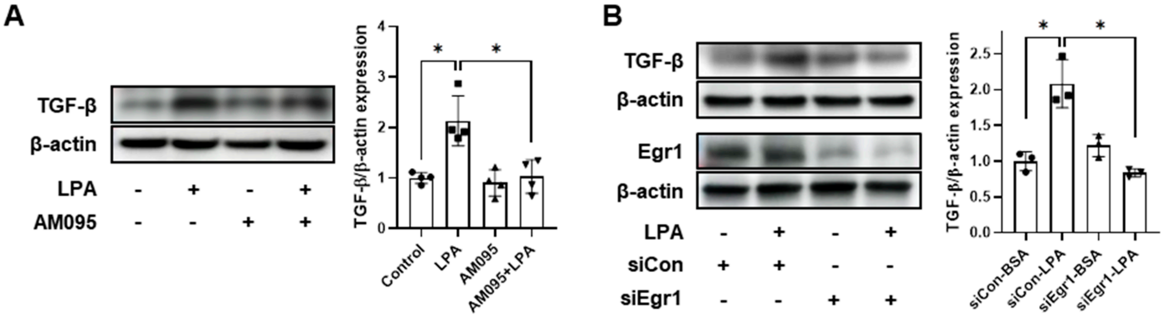

Figure S2. LPA induces TGF-β expression via Egr1 in E11 cells. (A) Differentiated E11 cells were treated with 5 µM LPA and 10 µM AM095 simultaneously for 6 h or with either AM095 or LPA alone. (B) Differentiated E11 cells were transfected with scrambled siRNA (siCon) or Egr1 siRNA (siEgr1) for 6 h, starved for 18 h, and then either treated with 5 µM LPA for 6 h or left unstimulated. The TGF-β protein level was analyzed via western blotting, quantified using the ImageJ software, and normalized to that of β-actin (*n* = 3 or 4). Data are represented as the mean ± SD. *: $p < 0.05$ via Kruskal–Wallis test followed by Dunn's test for multiple comparisons and Mann–Whitney U test for two specific groups.
